# Microplastics in early-onset carcinogenesis

**DOI:** 10.1093/carcin/bgaf093

**Published:** 2026-01-12

**Authors:** Anne M Bailey, Lorne J Hofseth

**Affiliations:** Department of Cell Biology and Anatomy, Cardiovascular Translational Research Center, School of Medicine, University of South Carolina, Columbia, SC 29206, United States; Department of Drug Discovery and Biomedical Sciences, College of Pharmacy, University of South Carolina, Columbia, SC 29208, United States

**Keywords:** microplastics, cancer, carcinogen, early onset, obesogen

## Abstract

Plastics have become integral to modern life, but their persistence has generated vast quantities of microplastics (MPs, <5 mm) and nanoplastics (NPs, <1 µm) that now contaminate food, water, air, and human tissues. Although not yet classified as carcinogens by the International Agency for Research on Cancer, accumulating experimental and epidemiologic evidence raises concern that MPs may contribute to cancer development. Global plastic production has risen from 2 megatons in 1950 to >450 megatons annually in 2022, leaving behind pervasive waste that fragments into MPs and NPs. These particles act as xenobiotics, carrying toxic additives and adsorbed pollutants, provoking oxidative stress, chronic inflammation, DNA damage, and microbiome disruption, all processes central to carcinogenesis. MPs have been detected in human cancers, and animal studies show tissue accumulation, fibrosis, and genomic instability following exposure. Importantly, the proliferation of plastics parallels a global rise in early-onset cancers (diagnosed before age 50), suggesting a possible, though unproven, temporal association. Individuals born after the 1950s plastic boom have experienced continuous MP exposure beginning *in utero*, potentially predisposing them to carcinogenic pathways later in life. In this review, we integrate human biomonitoring data, experimental findings, and clinical observations to evaluate the emerging hypothesis that chronic MP exposure contributes to cancer risk. While causality has not been established, the biological plausibility and mounting evidence underscore the urgent need for mechanistic and epidemiologic studies to clarify the role of MPs and NPs in cancer development. It also underscores an urgent need for research into causal pathways and preventive mechanisms.

## Graphical Abstract

**Graphical Abstract:**
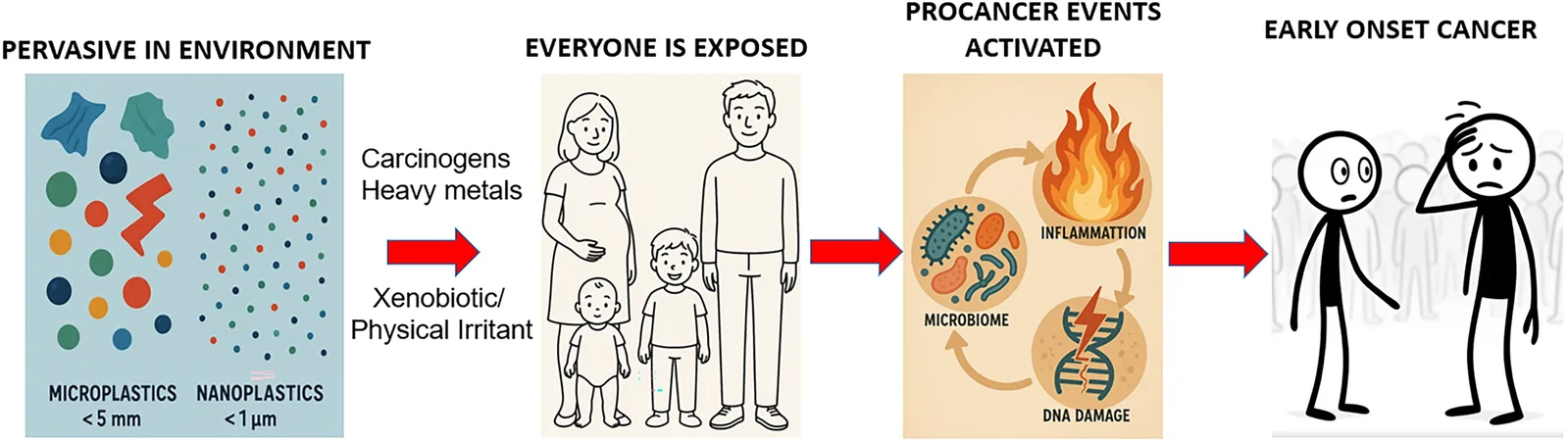

## 1. Introduction

Microplastics (MPs, <5 mm) and nanoplastics (NPs, <1 µm) are not officially classified as carcinogens by the International Agency for Research on Cancer (IARC) or comparable agencies, largely because existing evidence remains limited and indirect. Nonetheless, a growing body of observation and research raises significant cause for concern. Global plastic production has surged from 2 megatons (Mt) in 1950 to more than 450 Mt annually in 2022 [1]. This rapid increase has generated widespread and persistent waste that fragments into MPs and NPs, now infiltrating ecosystems and accumulating within humans.

MPs and NPs were first reported in human feces in 2019 [2] and have since been detected in blood, tissues, placentas, and even developing fetuses [3, 4]. Unfortunately, MPs are xenobiotics (foreign to the human body) that carry toxic additives and pollutants and provoke chronic inflammation, immune dysregulation, and oxidative stress. MPs and NPs can cause DNA damage, including oxidative lesions such as 8-oxo-deoxyguanosine, double-strand breaks marked by γ-H2AX, and endocrine and metabolic disturbances [5]. They also act as carriers for carcinogenic compounds such as polycyclic aromatic hydrocarbons, heavy metals, and endocrine disruptors [6]. Notably, MPs have been detected in human cancers [7], and animal studies demonstrate tissue accumulation accompanied by chronic inflammatory responses, including liver fibrosis, colitis, and brain deposition with neuroinflammation [3]. Collectively, these findings raise the concern that sustained MP exposure may confer long-term cancer risk.

Importantly, the rise in environmental plastic contamination coincides with a perplexing global increase in early-onset cancers (diagnosed before age 50). This temporal overlap has prompted hypotheses that MPs could represent a novel, previously unrecognized carcinogenic factor. Although no direct causal link to human cancer has been established, the biological plausibility, experimental data, and emerging epidemiologic evidence underscore the urgent need for systematic investigation. In this review, we analyze current evidence linking MPs and NPs to carcinogenic processes, with particular emphasis on early-onset cancers.

## 2. History of plastic use and microplastic emergence

The story of MPs is inseparable from the broader history of plastic production [8] (Table 1). Although the first synthetic plastics emerged in the early 1900s, large-scale manufacturing accelerated only after World War II. From the 1950s through the 2000s, global plastic output expanded exponentially as plastics became the backbone of modern consumer culture; valued for low cost, durability, and versatility across packaging, textiles, and countless household goods. That same durability that made plastics attractive also underpins their environmental and human threat: rather than biodegrading, discarded plastics fragment over time and generate vast quantities of persistent, potentially hazardous MPs. Indeed, MPs are now recognized as pervasive contaminants across nearly every environmental compartment. First reported off the Northumberland coast [9] and the Sargasso Sea [10] in the early 1970s, they have since been reported from the sea surface to deep-sea sediments, rivers, soils, sea ice, remote mountains, and the atmosphere [1]. The United Nations (UN) estimates that around 75–199 million tons of plastic are floating in the world’s oceans today [11]. Atmospheric fallout and monitoring studies have found MP fibers and particles in both urban and remote air samples, showing they can travel long distances and enter the human respiratory tract [12, 13]. Current best estimates place annual emissions of MPs (including leakage of particles and fragmentation products) on the order of tens of millions of tons per year, and under business-as-usual scenarios environmental burdens could increase substantially by 2040. In the USA, it is estimated that ∼22 000 tons of MPs are being released into the atmosphere annually [14]. With cumulative plastic waste now measured in billions of tons, the scale of MP pollution continues to intensify. This timeline has critical implications for human health: people born in the decades after the mid-20th century (particularly cohorts born after ∼1980) are the first generations to experience continuous, lifelong exposure to MPs from conception onward, and these exposures temporally overlap with rising incidence of some early-onset diseases, including certain cancers. The parallel trajectories of plastic proliferation and shifts in disease patterns underscore an urgent need for rigorous investigation into the possible role of MPs in human health. There is mounting evidence of widespread exposure and the remaining knowledge gaps (particularly for human health risk quantification) and calls for rigorous science and system-level policy responses alongside improved exposure and effect data.

**Table 1:** Timeline of plastics and MPs.

Year/period	Key event/discovery
1600 Bc—ancient	Use of natural “plastics” (amber, rubber, shellac, tortoiseshell) as durable organics in human material culture [8]
1862	Parkesine (first man-made plastic) marks the start of synthetic polymer era [15]
1907	Bakelite invented: Leo Baekeland developed the first synthetic plastic in 1907, and the patent for his invention was granted in 1909 [16]
1920s to 1930s	Rapid commercialization of major polymers (Polystyrene/PS, Polyvinyl Chloride/PVC, Polyethylene/PE, nylon); plastics become ubiquitous in consumer goods [17]
1950s	Rise of single-use/disposable plastics and “throwaway” culture, which accelerates production, use, and waste
Early 1970s	First scientific reports of synthetic fiber plastics in the ocean [9, 10, 18]
1980s	Recognition of large ocean accumulations (e.g. Great Pacific Garbage Patch [19]) lending to public awareness of the plastic crisis
1990	First experimental study showing marine species digest MPs. Here, the estuarine copepod “Eurytemora affinis” ingested and regurgitated inert particles (including plastic beads) [20]
1996	Early identification of MPs in personal care products as a waste source [21]
2003	First scientific reports of MPs found in aquatic species (fur seals) [22]
2004	Term “microplastics” popularized, a field-defining moment launching focused MP research [23]
2008	First scientific report of MPs found in shellfish (mussels) [24]
2010s (mid)	Key sources identified (textiles, tires, cosmetics, pellets, fragmentation); methodological advances (FTIR, Raman, py-GC-MS) and QA/QC begin [25, 26]
2018	Earliest peer-reviewed study to report MPs in consumables (bottled water, tap water, beer, table salt, and processed seafood) [27–29]
2019	MPs first detected in hot tea made from teabags [30]
2019	MPs first detected in humans (feces) [2]
2020	MPs first detected in baby formula [31]
2024	Thompson *et al* [8] synthesizes 20 years of evidence. This seminal paper concludes that we have enough evidence to agree that global action on MPs needs to take place and frames the UN Plastics Treaty as a crucial global initiative to address plastic pollution
2025	MPs first detected in hot brewed coffee [32]
2040 (projection)	Under business-as-usual, ∼710 million metric tons of plastic waste would cumulatively enter aquatic + terrestrial ecosystems between 2016 and 2040 [8, 33, 34]

## 3. Exposure to microplastics

Table 2 illustrates the extent of dietary exposure, showing that MPs occur in a wide array of consumables, ranging from beverages such as tea, coffee, milk, and bottled water to solid foods including fruits, vegetables, seafood, and table salt. Indeed, there is striking variability in both the “amounts” and “sources” of MPs, reflecting differences in how these items are produced, processed, and packaged.

**Table 2:** Common sources of human MP ingestion.

Consumable	Average MP load	Primary source	Reference
Hot coffee	∼21–63 MPs/L	Plastic pods	[32, 35]
		Processing	
Hot tea	∼28–7607 MPs/L	Plastic mesh teabag	[30, 32, 36, 37]
		Processing	
Cow’s milk	∼270 MPs/L	Processing	[38]
Soft drinks	∼10–22 MPs/L	Processing, packaging	[32, 36]
Energy drinks	∼12–41 MPs/L	Processing, packaging	[32]
Juices	∼20–48 MPs/L	Processing, packaging	[32]
Bottled water	Globally: ∼12–1570 MPs/L	Processing, packaging	[4, 27, 32, 36, 37, 39–43]
Tap water	Globally, ∼4.5–5.5 MPs/L	Environment	[4, 32, 36, 44]
Infant formula	∼4–49 MPs/100 g powder	Polypropylene bottle	[31, 36, 45]
Fruits and vegies	∼3–223 000 MPs/g	Environment	[36, 46, 47]
		Processing	
Seafood (shellfish)	∼20–40 MPs/g	Environment	[48]
Table salt	∼1386 MPs/kg	MPs in seawater	[49]

Beverages show some of the highest reported concentrations of MPs, particularly those that come into contact with plastic during preparation. For example, hot coffee brewed in plastic pods contains ∼21–63 MPs per liter [32, 35]. These particles originate from both the pod material and machine components exposed to heat and pressure. Similarly, hot tea demonstrates the widest range (∼28–7607 MPs per liter [30, 32, 36]) largely due to the type of teabag used. Plastic mesh teabags, when steeped in boiling water, can release thousands of particles per cup, whereas paper-based or biodegradable teabags release far fewer. These findings underscore how “processing and product design” directly influence MP contamination.

Cow’s milk, with an average load of ∼270 MPs per liter [38], highlights a less obvious contamination route: processing. Plastic tubing, filters, and storage containers used during pasteurization and packaging can shed MPs. Although milk is not an intuitively “plastic-rich” product, the mechanical handling and bottling processes introduce particles into the final product.

Commercial soft drinks, energy drinks, and juices exhibit moderate MP concentrations—roughly 10–48 MPs per liter [32, 36]—with contamination attributed primarily to packaging and bottling. These beverages are typically stored in polyethylene terephthalate (PET) bottles or aluminum cans with plastic liners, both of which are susceptible to degradation and abrasion during transport and storage. Repeated opening and closing of bottle caps also contributes to particle release.

The greatest variability occurs in bottled water, where global estimates are wide ranging (from a few to thousands of MPs per liter) [4, 27, 32, 36, 39–43]. Processing and packaging dominate as sources, since the bottling process involves high friction and pressure. In contrast, tap water generally contains far less MPs per liter [4, 32, 36, 44], with MPs arising mainly from environmental contamination in water distribution systems. The stark difference between bottled and tap water illustrates how packaging, rather than the water source itself, often determines MP burden.

Alarmingly, infant formula has been shown to contain 4–49 MPs per 100 g of powder [36, 45]. The particles predominantly originate from polypropylene bottles used for mixing and feeding. This finding is particularly concerning given infants’ small body mass and developmental vulnerability. Heating formula in plastic bottles accelerates MP release, highlighting the role of preparation methods in exposure.

Among solid foods, fruits and vegetables show the most extreme variability, from as low as 3 MPs per gram to an astonishing 223 000 MPs per gram [36, 46, 47]. This wide range reflects both environmental contamination—such as atmospheric deposition, irrigation with contaminated water, and the use of plastic mulch in agriculture—and processing and packaging practices after harvest. These findings suggest that plants can absorb or accumulate small plastic particles from soil and water, particularly in root crops and leafy greens, making produce an emerging vector of human exposure.

Seafood (shellfish) averages ∼20–40 MPs per gram [48], consistent with the known accumulation of MPs in marine organisms. Shellfish ingest particles present in seawater, which then remain in their tissues and digestive tracts. Because humans consume these animals whole, seafood serves as a direct route of MP transfer from marine ecosystems to humans.

Table salt, averaging ∼1386 MPs per kilogram [49], exemplifies contamination via environmental exposure. Since sea salt is derived from evaporated seawater, MPs suspended in marine environments become concentrated in the final product. Even though daily salt intake is small, the per-mass concentration is high enough to make this a measurable contributor to cumulative MP exposure.

When comparing these values collectively, a clear pattern emerges: processing, packaging, and environmental contamination are the dominant pathways through which MPs enter the human diet. Beverages and liquid foods tend to acquire MPs primarily from packaging materials, while solid foods such as produce and seafood reflect environmental sources. In almost every case, the level of MP contamination correlates more strongly with the degree of human handling and contact with plastic than with intrinsic food composition.

Despite these insights, the variability across studies highlights major methodological gaps. Differences in sampling, particle size detection, and analytical techniques (e.g. Fourier Transform Infrared/FTIR vs. Raman spectroscopy) lead to inconsistent results. Furthermore, most measurements focus on MPs >20 µm, potentially underestimating the true burden of smaller, more biologically active NPs. Concentrations vary from a few particles per liter to hundreds of thousands per gram, depending on the consumable and its interaction with plastic materials. Processing and packaging are primary sources for beverages and dairy, while environmental exposure dominates in produce, seafood, and salt. These findings underscore that dietary ingestion of MPs is now unavoidable and emphasize the urgent need for standardized analytical methods, safer packaging alternatives, and continued investigation into the health implications of chronic exposure.

## 4. Rise of early-onset cancers and the microplastic hypothesis

Early-onset cancers (those diagnosed before age 50) have been rising worldwide since the early 1990s, even as many cancers in older adults decline due to improved prevention and screening. This troubling trend has been documented in colorectal cancer [50, 51], breast cancer [51], pancreatic cancer [51]}, and uterine/endometrial cancer [51]. Rising incidence has also been reported in early-onset kidney cancer (England [52]; USA [51], gastric cancer (USA [53]), thyroid cancer (GLOBCAN database [54]), lung cancer (China [55]), and prostate cancer (Global [56]).

Several consistent features of early-onset cancers help narrow potential causes (Table 3): (i) long-term trend: incidence and mortality have been climbing since the 1980s; (ii) geography: while global, the rise is most pronounced in East Asia and high- to middle-income regions [57]; (iii) distinct biology: early-onset cancers often show unique molecular and pathological signatures compared to late-onset cases; and (iv) mechanistic pathways: dysbiosis, chronic inflammation, immune disruption, DNA damage, and metabolic/epigenetic alterations appear central to their development. Because cancer typically develops decades after exposure, the driving factor must have emerged earlier. Thus, an exposome element rising sharply between the 1950s and 1970s is a logical candidate. Plastic production fits this timeline, and children have a high exposure. Global plastic output grew from ∼1.5 million metric tons in 1950 to 105 million metric tons by 1990 [1, 58].

**Table 3:** Case for an association between early-onset cancers and MP exposure.

Dimensions	Evidence for early onset cancers	Evidence for exposure	Link?
Trends/epidemiology	Rise in early-onset cancers since the late 1980s [50]	Global rise in plastic production since 1950s and 1960s	Temporal trend
Geography	Incidence especially rising in East Asia and middle- to high-income areas (e.g. North America, Australia, Europe)	Highest exposure in developing regions, but growing evidence that it is widespread (lack of studies quantifying abundance)	Partial geographic overlap but no proven correlation. Identifying overlapping hotspots will be valuable information (yet to be done)
Exposure	MPs are everywhere and are found in >90% of human samples, including cancer tissues tested [59]	MPs ingested via food, water, air, and soil	Animal studies link exposure to carcinogenesis [60]. No human studies
Biological mechanisms	Early-onset cancers show a unique pathology (e.g. poorly, differentiated, mucinous, or signet-ring cell morphology) [61]. Often have a strong inflammatory component	Animal studies show MPs cause inflammation and can cause a mucinous pathology [62, 63]	MPs can cause some of the same pathologies seen in EOC

Linking plastic production to MP exposure is more complex. Exposure is highest in developed countries, possibly due to the abundance of bottled water, baby formulas, and teabags (Table 2). However, disadvantaged groups within these regions face the greatest risk, as they are also more reliant on ultra-processed foods, a major source of MPs. In developing countries, underserved populations may similarly bear both high exposure and high cancer risk. Indeed, MPs have been detected in early-onset cancers, including colorectal [7, 64] and breast cancers [59].

Emerging evidence supports a biological link between MP exposure and early-onset cancers. MPs and NPs induce reactive oxygen species (ROS), lysosomal dysfunction, and chronic inflammation; all well-established drivers of oncogenesis [65]. In the gut, MPs disrupt the mucus barrier, alter microbiome homeostasis, and increase intestinal permeability, fueling pro-carcinogenic inflammation. These changes are increasingly associated with early-onset gastrointestinal cancers [66]. MPs also serve as carriers for toxins and pathogens, compounding genotoxic stress and immune dysregulation. Animal studies of “plasticosis,” a fibrosis-like pathology caused by chronic plastic exposure, further illustrate how MPs may reshape tissues and create a tumor-permissive environment [67, 68]. Taken together, these findings suggest that MPs promote the inflammatory, microbial, and stromal footprints increasingly recognized as hallmarks of early-onset cancers. This could explain why these cancers often display distinct biological profiles compared to their late-onset counterparts.

Of course, early-onset cancers are multifactorial and linked to obesity, antibiotic use, and dietary patterns. Yet the timing, geography, and molecular signatures of the disease strongly implicate MPs as a plausible contributor. The rapid rise of early-onset cancers over the past three decades parallels the exponential global production and environmental dissemination of plastics, suggesting a shared temporal trajectory. Unlike most traditional carcinogens, MPs represent a continuous, low-dose exposure that begins prenatally and persists throughout life, affecting multiple organ systems. As mentioned, MPs and NPs have been detected in tissues and blood, confirming their early and systemic bioavailability. This chronic exposure may “prime” developing tissues through oxidative stress and immune imprinting, increasing susceptibility to oncogenic transformation later in life.

## 5. Mechanisms linking microplastics to carcinogenesis

Cancer develops when cells accumulate mutations and adapt their surrounding environment to support uncontrolled growth. The hallmarks of cancer [69, 70], such as unchecked proliferation, evasion of cell death, chronic inflammation, and genome instability, offer a framework for understanding how new environmental stressors may drive tumorigenesis. MPs, once considered biologically inert, are now recognized as potential contributors at multiple stages: they can trigger DNA damage through oxidative stress, sustain inflammation and immune disruption, disturb the gut microbiome, and promote metabolic dysfunction such as obesity—all factors known to accelerate cancer risk. Importantly, these mechanisms overlap with trends seen in the rising incidence of early-onset cancers, where environmental exposures are suspected to play a larger role than inherited risk. By shaping tissues into pro-carcinogenic environments, MPs may represent a hidden but significant contributor to cancer development earlier in life.

### 5.1. Chronic inflammation and immune dysregulation

MPs have emerged as significant environmental pollutants capable of disrupting human immune and inflammatory processes, thereby influencing disease progression. Due to their small size, variable surface charge, and chemical persistence, MPs can cross biological barriers and accumulate in tissues, where they interact with immune cells and disturb homeostasis. Experimental and clinical studies show that MPs induce oxidative stress and activate multiple pro-inflammatory signaling cascades.

Initially, MP exposure provokes chronic inflammation by stimulating the production of ROS and activating inflammatory mediators such as the NLRP3 inflammasome [71]. This activation promotes the release of interleukin-1β, interleukin-6, tumor necrosis factor alpha (TNF-α), and transforming growth factor beta [72]. The sustained elevation of these cytokines drives persistent inflammation, impairs immune surveillance, and fosters a tumor-promoting microenvironment.

As inflammation becomes chronic, it progressively activates oncogenic signaling pathways (including Nuclear Factor kappa‑light‑chain‑enhancer of activated B cells/NF-κB, Phosphoinositide 3-Kinase/PI3K, v-Akt murine thymoma viral oncogene homolog/Akt, Mechanistic Target of Rapamycin/mTOR, Wnt/β-catenin, and p53) thereby facilitating tumor initiation, growth, and metastasis [73]. MPs also cause mitochondrial damage [74], further amplifying oxidative stress and reinforcing these inflammatory feedback loops.

Evidence from human studies supports this mechanistic framework. A pivotal investigation identified 15 distinct types of MPs in human stool, with significantly higher concentrations in patients with inflammatory bowel disease compared to healthy individuals [75]. In the gastrointestinal tract, MPs promote ROS generation, epithelial barrier dysfunction, and gut microbiota dysbiosis, all contributing to intestinal inflammation and immune dysregulation. MP-induced alterations in microbiota composition (including shifts in the Firmicutes-to-Bacteroidetes ratio [76]) have been linked to oxidative stress, immune activation, and chronic intestinal inflammation.

Given that chronic intestinal inflammation is a well-established risk factor for colorectal cancer in Inflammatory Bowel Disease (IBD) patients, these findings suggest that MP exposure exacerbates IBD severity and accelerates the inflammatory-to-carcinogenic transition. Collectively, these data indicate that MPs act not only as environmental contaminants but also as bioactive modulators of immune signaling. By sustaining NF-κB- and MAPK-mediated cytokine release, altering T-helper-cell differentiation toward Th17 and Treg phenotypes, and promoting microbiota-driven dysbiosis, MPs intensify chronic gut inflammation and potentiate oncogenic transformation [77].

### 5.2. DNA damage and genomic instability

MPs and NPs can induce cellular stress and DNA damage through direct and indirect mechanisms, potentially contributing to carcinogenesis. A major pathway is oxidative stress, where excess ROS overwhelm antioxidant defenses, causing oxidative base lesions (e.g. 8-oxoguanine) and strand breaks. In zebrafish embryos, microinjection of 20 nm polystyrene (PS)-NPs into the yolk sac led to accumulation in the brain and elevated oxidative DNA lesions [78]. Similarly, ROS-induced DNA damage markers like 8-OHdG increased in NP-exposed neural tissues [78]. In human epithelial cell cultures, smaller and more concentrated PS-MPs caused stronger cytotoxic effects (excessive ROS, γ-H2AX foci, p53-mediated cell-cycle arrest, and apoptosis), while antioxidants mitigated these responses [79].

In *Drosophila*, oral exposure to polyethylene MPs (1–10 mg/ml) impaired motor functions and induced oxidative stress, shown by increased ROS, decreased Superoxide Dismutase (SOD), and elevated Glutathione-S-Transferase (GST) activity [80]. These flies also upregulated stress and apoptosis genes (p53, reaper), indicating activation of conserved cell-death pathways [80]. In mice, chronic MP ingestion worsened infection-driven liver injury, enhancing p53/p21 signaling and a pro-carcinogenic transcriptomic profile with increased Sall2 expression [81].

Chronic NP exposure can also drive genotoxic and transformative changes. Human bronchial epithelial cells continuously exposed to 50 μg/ml PET NPs for 30 weeks accumulated DNA damage, grew in soft agar, and acquired invasive traits [82]. Transcriptomics showed upregulation of oncogenes and lung cancer–associated genes [82]. In kidney organoids, PS-MPs (1.25–20 µg/ml) disrupted development through ROS-driven apoptosis (elevated Bax, reduced Bcl-2, and activated caspases), resulting in loss of nephron progenitors and impaired differentiation [83].

A systematic review reported that about half of NP genotoxicity assays (29/60) detected DNA strand breaks, oxidative lesions, or micronucleus formation [84]. PS nanoparticles were the most studied, and surface modifications (amino or carboxyl groups) increased DNA damage potential [84]. Immune cells appeared more sensitive than epithelial cells, while particle size showed no consistent effect. Limited *in vivo* studies have observed increased DNA strand breaks in blood and brain tissues following NP exposure [84]. Overall, MPs/NPs generate ROS, activate p53, and disrupt cell survival signaling, mechanisms plausibly linking chronic exposure to cancer pathways.

Although mechanistic data support biological plausibility, evidence remains insufficient to conclude that MP/NP exposure causes cancer in humans. Most findings are from *in vitro* or short-term *in vivo* studies. While oxidative DNA damage and micronucleus formation occur via ROS, direct evidence of mutations in cancer driver genes is sparse, and it is unclear whether antioxidants fully prevent NP-induced damage. Some studies show antioxidants like *N*-acetylcysteine reduce NP-induced DNA lesions [84]. However, NPs may also impair DNA repair. For example, PS-NP exposure (100 µg/ml) in Caco-2 cells suppressed genes for base excision and double-strand break repair (e.g. OGG1, LIG1/3, PARP1/2, XRCC1, ATM, ATR, BRCA1, RAD51, XRCC4, and TP53BP1) [85]. Likewise, 20 nm amine-modified PS-NPs directly bound and cleaved DNA *in vitro*, unlike carboxylated or larger particles [86].

Emerging evidence also suggests NPs may impair genomic maintenance. In both Caco-2 cells and *C*aenorhabditis *elegans*, PS-NPs downregulated base excision repair genes, whereas zebrafish embryos exhibited compensatory upregulation of oxidative defense and BER genes [87]. Few studies have tested whether mitigating oxidative stress prevents NP-induced genotoxicity; initial antioxidant data are promising but limited. In summary, while oxidative DNA damage and stress signaling clearly occur following MP/NP exposure, definitive evidence linking these effects to human cancer is lacking. Long-term *in vivo* and epidemiological studies are needed to establish causality.

### 5.3. Epigenetic alterations

Emerging studies suggest that MPs, once regarded as inert pollutants, can disrupt epigenetic regulation and thereby influence disease susceptibility. Experimental models show that exposure to PS-NPs induces DNA hypomethylation [88], while MPs also drive aberrant DNA methylation, histone modifications, and dysregulation of non-coding RNAs [89]. Beyond DNA methylation, MP exposure alters miRNA expression and their target genes, which are linked to immune responses, apoptosis, protein interactions, and cellular stress pathways [90]. For instance, PS-NPs fed to mice accumulate in the intestine and compromise intestinal integrity by altering miR-501-3p and miR-700-5p expression. This downregulation reduces ZO-1 and MUC-13, ultimately increasing gut permeability [91]. Although other studies have reported MP- and NP-induced changes in miRNA expression, most experiments have been conducted in non-human vertebrates such as fish. For example, polyethylene MPs caused muscle fiber damage and oxidative stress in carp while reducing miR-21 expression. This suppression activated the Interleukin-1 Receptor–Associated Kinase 4/IRAK4/NF-κB pathway, resulting in inflammation, endoplasmic reticulum stress, and apoptosis in muscle tissue [92]. Taken together, these findings clearly demonstrate that MPs and NPs can interfere with epigenetic machinery; however, data from human studies remain scarce, and translational research is limited. Indeed, such disruptions underscore the epigenetic plasticity induced by MPs, with potential consequences for tumor suppressor silencing, oncogene activation, and the promotion of carcinogenic pathway. Notably, MPs’ ability to adsorb and transport xenobiotics such as heavy metals and pesticides further amplifies their toxicity, delivering multiple insults to the epigenetic landscape.

### 5.4. Reproductive toxicity

Reproductive toxicity has emerged as a central outcome of MP-induced epigenetic perturbations [93]. Early-life exposure to reproductive toxicants is linked to elevated cancer risk. For example, endocrine-disrupting chemicals disrupt organogenesis, alter tissue architecture, and sensitize tissues to hormones, thereby predisposing to carcinogenesis. Epigenetic reprogramming (including DNA methylation and gene expression changes) may further promote malignant transformation, with possible transgenerational effects. Mutagens acting during critical developmental windows exacerbate risk by inducing mutations in rapidly dividing cells. A classic example is diethylstilbestrol (DES). Used between the 1940s and early 1970s to prevent miscarriage, DES is now recognized as a transplacental carcinogen: *in utero* exposure confers a ∼40-fold increased risk of clear cell adenocarcinoma of the vagina or cervix [94]. DES-exposed women also show higher rates of reproductive tract abnormalities, infertility, adverse pregnancy outcomes, and a modestly increased breast cancer risk. This case underscores how *in utero* exposure to reproductive toxicants can produce lifelong carcinogenic consequences through endocrine disruption, abnormal tissue development, and epigenetic reprogramming. Although research directly linking MPs to early-onset cancers is limited and in its infancy, substantial evidence indicates that MPs impair reproductive health. Photo-aged MPs alter germline histone methyltransferase expression, leading to apoptosis and defective DNA damage responses [95]. Low-dose PS-MPs impair oocyte maturation through HDAC3 insufficiency and H4K16 hyperacetylation [96], while PS-NPs accumulate in testes, upregulating steroidogenic enzymes and disrupting testosterone synthesis [97]. Transgenerational studies in *C. elegans* reveal MP-induced histone methylation changes (H3K4me2, H3K9me3) and gene upregulation (set-30, met-2) [98], while maternal NP exposure produces heritable H3K4/H3K9 methylation effects [99]. These outcomes, driven by oxidative stress, inflammation, mitochondrial dysfunction, and apoptosis, represent a major threat to reproductive health.

The overlap between reproductive toxicity and early-onset cancer is striking. MPs and other EDCs disrupt development during sensitive windows (*in utero*, childhood, and puberty), when epigenetic programming and hormone regulation are highly vulnerable. Such perturbations impair fertility while predisposing tissues to malignant transformation. Epidemiological and experimental studies link early-life exposures to reproductive disorders and cancers such as colorectal and breast malignancies. Stem cells appear central to this process: oxidative stress, inflammation, and epigenetic reprogramming impair germline and somatic stem cell function, reducing regenerative capacity while promoting oncogenesis. MPs, for instance, disrupt intestinal stem cells [100], potentially contributing to early-onset colorectal cancer. Together, these findings reveal a mechanistic link between MPs, reproductive toxicity, and cancer susceptibility. Epigenetic instability in germ cells and pluripotent stem cells not only impairs fertility but also increases the probability that aberrant DNA methylation patterns and histone modifications are transmitted to offspring or become fixed within somatic lineages. By perturbing developmental epigenetic programming during sensitive windows, MPs may predispose progeny to tumorigenesis at earlier stages of life. This mechanism could contribute to the observed global rise in early cancers [101]. Accordingly, MPs should be considered not merely as environmental contaminants but as potent epigenetic disruptors, with reproductive toxicity serving as a mechanistic bridge between transgenerational disease risk and the increasing incidence of early-onset malignancies.

### 5.5. Microbiome disruption

A comprehensive review of the impact of MPs on the human microbiome is provided by Thin *et al.* [102]. MPs enter the human body mainly through ingestion, inhalation, and skin contact. Evidence indicates that MPs consistently disrupt the gut microbiome by reducing beneficial bacteria such as *Lactobacillus*, *Blautia*, and *Parabacteroides*, while enriching pathogenic species including Enterobacteriaceae, *Escherichia-Shigella*, and *Bilophila* [102]. These shifts cause gut dysbiosis, often reflected in an altered Firmicutes/Bacteroidetes ratio associated with obesity, inflammatory bowel disease, and metabolic disorders. MPs generally reduce α-diversity and richness, a hallmark of chronic conditions such as inflammatory bowel diseases and obesity. At the metabolic level, MPs impair short-chain fatty acid production [103], reducing protective metabolites like butyrate and propionate. This compromises gut barrier integrity and promotes inflammation. MPs also alter aryl hydrocarbon receptor activity [104], weakening immune regulation and disrupting host metabolism. Collectively, these changes contribute to intestinal disease, systemic inflammation, metabolic syndrome, and possibly rising rates of early-onset cancers. Animal models further demonstrate the microbiome-disrupting effects of MPs and NPs. Long-term exposure to PS microspheres (PS-MS) in high-fat diet mice worsens obesity, liver disease, and systemic inflammation, with fecal microbiota transplantation confirming dysbiosis as the driver [105]. PS-NPs increase intestinal permeability, reduce tight junction proteins, and trigger ROS production, with protective but limited compensatory pathways. Real-world Polyvinyl Chloride/PVC fragments containing endocrine disruptors induce inflammation, oxidative stress, and gut microbiota shifts, particularly in a colitis model [106, 107]. In aged mice, PS-MPs exacerbate dysbiosis and metabolic changes, increasing vulnerability to age-related disease [108].

Of particular concern, young mouse studies show that MPs and NPs disrupt the microbiome during critical developmental periods. Co-exposure to MPs and tetracycline caused severe barrier dysfunction, reduced probiotics, enriched pathogens, and spread antibiotic resistance and virulence genes [109]. Early-life exposure in pregnant mice revealed size-dependent biotoxicity, with smaller PS-NPs crossing placental and mammary barriers, accumulating in organs, and causing more severe metabolic disruption than larger PS-MPs [110]. Taken together, MPs and NPs consistently disturb gut microbial balance, impair short-chain fatty acid production, and promote inflammation and barrier dysfunction. The consequences extend beyond intestinal health, contributing to systemic disease and possible cancer risk. Critically, evidence from young mouse models highlights early-life exposure as a particularly vulnerable window with long-lasting implications.

### 5.6. Obesity and metabolic dysfunction

Growing evidence indicates that MPs and NPs may function as environmental “obesogens,” driving metabolic dysfunction through gut dysbiosis, systemic inflammation, and endocrine disruption. Epidemiological studies, for instance, associate frequent take-out food consumption with elevated MP ingestion, gut microbiota alterations, and higher body mass index in young adults [111]. Experimental models corroborate these findings, showing that MPs induce glucose intolerance, visceral fat accumulation, and insulin resistance, accompanied by macrophage infiltration and TNF-α upregulation in adipose tissue [112]. Maternal exposure research further reveals transgenerational effects: offspring of NP-exposed mothers exhibit increased adiposity and altered gut microbiota, suggesting *in utero* metabolic programming [113]. Likewise, gestational NP exposure promotes obesity development in offspring by driving the whitening of brown adipose tissue [114]. Together, these studies support the notion that MPs and NPs exacerbate metabolic syndrome pathways, contributing not only to obesity but also to related conditions such as Type 2 diabetes and fatty liver disease. In animal models, MP exposure accelerates the progression of nonalcoholic fatty liver disease to steatohepatitis and even liver cancer, likely via oxidative stress and dysregulated hepatic lipid metabolism [115]. In mice fed a high-fat diet, chronic low-dose PS-MP exposure intensified nonalcoholic fatty liver disease progression, amplifying hepatic inflammation and oxidative stress; key mechanisms linking obesity to liver cancer risk [116]. This evidence aligns with the exposome framework, underscoring that environmental contaminants, including MPs, must be considered alongside diet and lifestyle in shaping obesity trajectories and early-onset cancer risk. Collectively, findings support a model in which MPs act both upstream; by disrupting metabolism and promoting adipogenesis (and downstream) by reshaping tissue microenvironments to predispose cells to tumor initiation and progression. Importantly, MPs are not merely passive contaminants but active disruptors of host metabolism and tissue homeostasis, amplifying chronic low-grade inflammation. This dual action on systemic metabolic regulation and local tissue environments, offers a mechanistic explanation for their links to both obesity and cancer, positioning MPs as emerging global health threats.

## 6. Conclusions and the road ahead

Although MPs and NPs are not currently classified as carcinogens by IARC or other regulatory authorities, growing evidence suggests they may contribute to cancer development (Fig. 1). Far from biologically inert, MPs and NPs have been shown to accumulate in human tissues, where they trigger oxidative stress, chronic inflammation, and direct DNA damage. They can disrupt gut and epithelial barriers, alter the microbiome, interfere with metabolic and immune signaling, and serve as carriers for known carcinogens such as heavy metals, PAHs, and endocrine disruptors. These mechanisms overlap with established cancer pathways, raising concern that chronic exposure could create a tumor-permissive environment.

**Figure 1:**
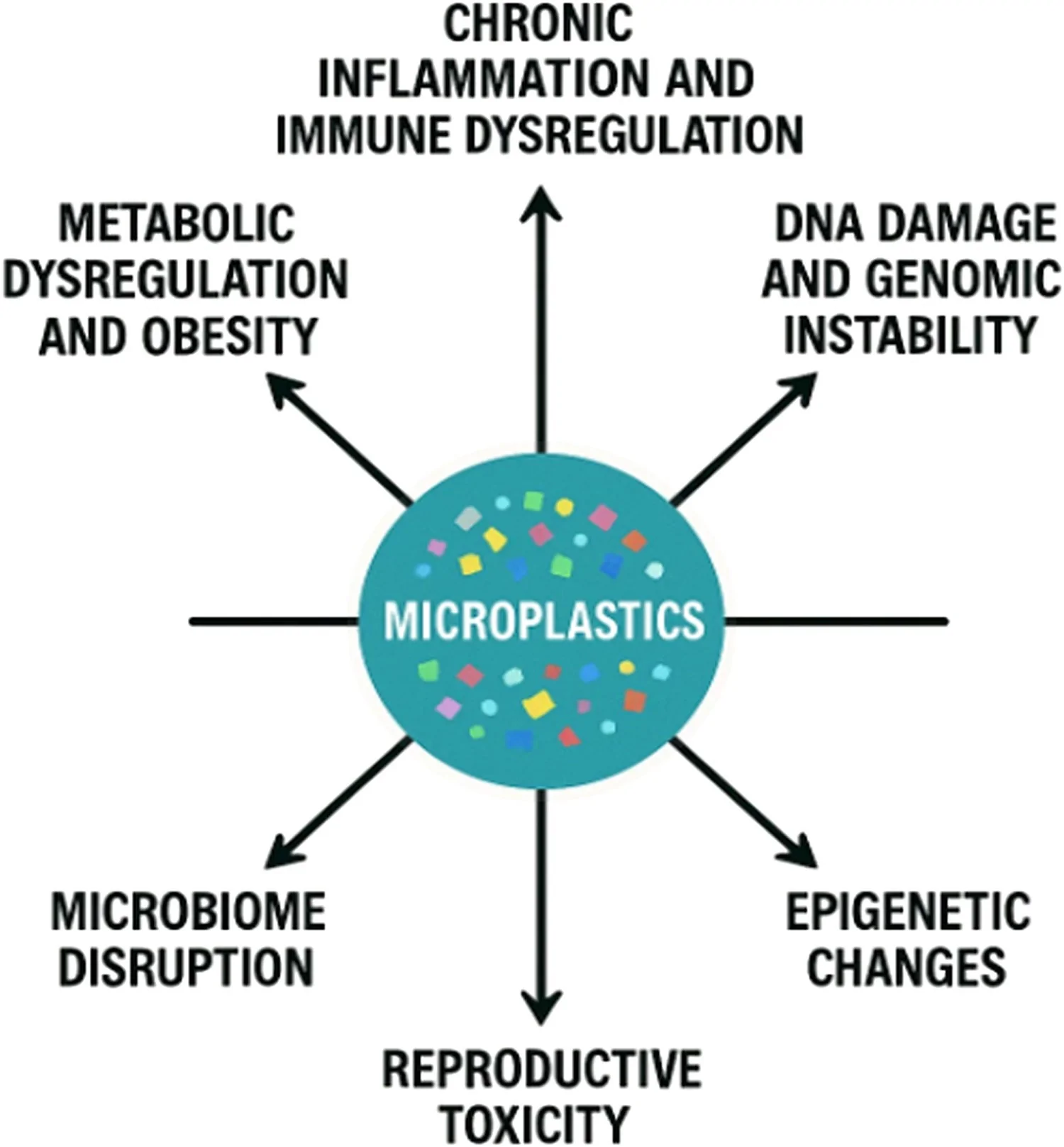
MPs cause chronic inflammation and immune dysregulation, DNA damage and genomic instability, epigenetic changes, reproductive toxicity, microbiome disruption, metabolic dysregulation, and obesity.

Applying Bradford Hill’s framework [117] highlights both the strengths and limitations of the current evidence (Table 3). Biological plausibility is well supported: MPs and NPs clearly provoke DNA damage, oxidative stress, and endocrine disruption, while also acting as vectors for carcinogens. Temporality is only partially met. Rates of early-onset cancers have risen since the 1980s, coinciding with the first generations exposed to MPs from conception [51], but longitudinal human evidence is lacking. Strength of association is not yet demonstrated, as epidemiological data remain sparse, though MPs have been detected in colorectal and breast cancer tissues, but no large cohort or case-control studies quantify cancer risk relative to exposure. A dose–response relationship is supported in animal and *in vitro* studies, where higher concentrations of MPs and NPs produce more DNA damage and inflammation, but human data are sparse. Consistency is partially met: multiple labs confirm MPs accumulate in tissues and induce genotoxicity in animals, and MPs are now detected in human biospecimens worldwide, yet direct cancer outcomes are inconsistent. Specificity is not met, as MPs cause diverse systemic effects beyond cancer, acting more as broad risk amplifiers. Finally, experimental evidence is strong at the preclinical level: rodent and cell studies confirm MPs cause DNA damage, fibrosis, and tumor-supportive environments, though no long-term animal or human studies have yet confirmed tumor formation directly caused by MPs.

Taken together, MPs and NPs meet some, but not all, of Bradford Hill’s criteria for causation. They are biologically plausible carcinogens, supported by experimental data, but the absence of strong epidemiological evidence and long-term human studies prevents a definitive conclusion. The parallel rise of MP exposure and early-onset cancers in younger generations underscores the urgency of further research. Large-scale cohort studies, improved biomarkers of exposure, and mechanistic work in human models are needed to determine whether MPs and NPs are not just contributors to, but actual causes of, cancer. Until then, the question of carcinogenicity remains unsettled, but the mounting signals demand closer scrutiny; particularly for children and young adults, who may bear the greatest burden of lifelong exposure.

## Data Availability

No new data were generated or analyzed in support of this research.
